# Dangerous Membranes: Viruses That Subvert Autophagosomes

**DOI:** 10.1016/j.ebiom.2014.11.015

**Published:** 2014-11-27

**Authors:** William T. Jackson

**Affiliations:** Department of Microbiology and Molecular Genetics and Center for Infectious Disease Research, Medical College of Wisconsin, 8701 Watertown Plank Road, Milwaukee, WI 53226, United States

In this issue of *EBioMedicine*, Christian Münz and colleagues show that Epstein–Barr Virus (EBV) induces an accumulation of membranes related to the cellular autophagy pathway, and that an autophagic marker can be found in EBV particles (Nowag et al., 2014). This suggests that autophagic membranes participate in generation of the viral envelope. This suggests new avenues for inhibiting gamma-herpesvirus formation and the possibility of antigenicity from autophagic markers on virions. To understand the importance of this finding, we need to examine autophagy and its relationship to viruses.

Autophagy is a constitutive pathway of protein, lipid, and organelle breakdown that serves to maintain cellular homeostasis. During times of stress, starvation, or developmental programming, autophagy increases in cells. Autophagy is marked by the generation of unique double-membraned vesicles that encompass cytoplasmic cargo for degradation. The pathway is regulated at several stages, including formation of the autophagosome and the stepwise maturation of the organelle, by vesicle fusion, into an acidic, degradative autolysosome. This regulatory precision means that autophagosomes can form without maturing into degradative vesicles, such that the presence of autophagic vesicles does not necessarily mean active autophagy.

In the early days of the virus-autophagy field, two non-exclusive theories were advanced about the relationship between infection and autophagosome formation. First, it was suggested that autophagy acts as a direct, physical component of innate immunity, degrading nascent virions and viral components to inhibit infection. Certainly this is true for some viruses; however, we now know that this is far from a universal phenomenon, and that autophagy is often triggered by the pathogen as a pro-viral pathway. The second theory was that the surface autophagosomes serve as a physical location or substrate for viral processes; notably, in the case of RNA viruses, RNA replication has been reported to take place on the autophagosome surface (Richards et al., 2014). There is ample evidence that this does in fact occur, but recent work has demonstrated that autophagosomes serve purposes later in viral life cycles, including roles in physical assembly, maturation, and cellular exit of virions, for both RNA and DNA viruses. The Münz group's work highlights the broad application of this viral strategy (Nowag et al., 2014).

In 2005, the lab of Karla Kirkegaard reported that regulating levels of cellular autophagy altered the levels of extracellular poliovirus prior to cellular lysis. This phenomenon was termed **A**utophagic exit **W**ith **O**ut **L**ysis, or AWOL (Jackson et al., 2005). This led to a hypothesis that autophagy could be related to a secretory pathway, and that the virus was taking advantage of said pathway. Although there was, at the time, no evidence that such a pathway existed, it was later shown that autophagy does feed into non-canonical cellular secretion. This unconventional secretory mechanism, first identified in yeast and amoebae, is not mediated by ER signal sequences and appears to be a relatively minor pathway (Pfeffer, 2010). However, the existence of this secretory mechanism does reveal that autophagy can be a pathway of cell exit. A major topology problem remains: namely, how might a virus transverse multiple lipid bilayers (two on the autophagosome, and one on the plasma membrane) to be released from cells without a surrounding membrane? Recent papers have indicated that picornaviruses, including Hepatitis A Virus and Coxsackievirus B3, are often released in membranous vesicles and are therefore not purely non-enveloped. In the case of Coxsackievirus, the membrane surrounding extracellular viral particles contains markers of autophagy (Robinson et al., 2014). This suggests that fusion of the outer autophagic membrane with the plasma membrane could release a single-membraned packet of virions. Ironically, then, a role for autophagic membranes in viral “envelope” formation was first identified in supposedly non-enveloped viruses. Recent data have demonstrated a similar role for autophagy in an enveloped RNA virus family, with the autophagy pathway used by Flaviviruses for both particle maturation and release.

A pair of recent papers in the *Journal of Virology* introduced the idea of a functional relationship between the autophagy pathway and EBV. Hung et al. showed that the EBV transcription factor RTA activates transcription of key autophagy genes, and that inhibition of autophagy reduces lytic progression of EBV (Hung et al., 2014). In a study published a week later, Granato et al. showed that EBV subverts the autophagy machinery to promote its own replication while inhibiting the process of autophagic degradation (Granato et al., 2014). The latter is not an unusual strategy; the maturation of the autophagosome into a degradative autolysosome is tightly regulated. Coxsackievirus B3 has a similar relationship with the machinery of autophagy, generating autophagic structures without inducing degradation of their cargo (Kemball et al., 2010). Therefore, the presence of autophagic membranes does not necessarily indicate the presence of active autophagy. Until now, however, the role of these membranes in EBV replication was not understood.

In this issue, Münz and colleagues show that autophagic membranes are stabilized during EBV infection, which fits with the findings of Granato et al. that the pathway is activated but degradative throughput is blocked (Nowag et al., 2014). Inhibition of autophagic pathways increases EBV DNA levels in the cytosol while decreasing infectious virus levels, indicating a role for autophagosomes in virion packaging and assembly. The authors go on to provide direct evidence that autophagosome membranes are incorporated into traditional viral envelopes, identifying a key autophagy marker, the lipidated form of cellular LC3, in EBV particles. The authors come to the conclusion that EBV subverts the machinery of autophagic membrane formation to form its own second viral envelope.

Several questions remain. It is tempting, at first thought, to imagine that diverting a non-canonical secretory pathway for particle assembly allows EBV to avoid the major secretory pathways used in immune responses. However, there is ample evidence that the autophagy pathway actually feeds into multiple immune response pathways, particularly the MHC Class II pathway, and late-expressed EBV proteins are presented by MHC Class II in an autophagy-dependent manner (Puleston and Simon, 2014). It appears that the abundant and flexible source of membranes provided by the autophagy machinery is usurped for pro-viral purposes with little regard for their normal cellular function. The trade-off for using autophagic membranes, of course, seems to be that virus-derived peptides may then be presented on MHC molecules. Presumably this recognition by the immune system is worth the not-yet-understood advantages provided by autophagic membranes.

The findings presented in this issue make even more of a case for understanding the relationship between viruses and autophagy, and in particular the advantages conferred by subverting the autophagic pathway. It is certainly possible that proteins of the autophagy pathway will make useful targets for therapeutics, to inhibit assembly of EBV and other viruses. It is even possible that the presence of lipidated LC3 on viral membranes could play a role in immune evasion, causing EBV particles to be recognized as part of the host — perhaps as autophagy-derived exosomes, recently shown to be part of broad anti-viral responses (Delorme-Axford et al., 2013). In any case, the next several years are certain to bring an increased focus on understanding the myriad advantages of autophagic subversion by even more pathogens.

## Conflict of interest

The author declares no conflict of interest.
